# Explainable CNN–Radiomics Fusion and Ensemble Learning for Multimodal Lesion Classification in Dental Radiographs

**DOI:** 10.3390/diagnostics15161997

**Published:** 2025-08-09

**Authors:** Zuhal Can, Emre Aydin

**Affiliations:** Computer Engineering Department, Engineering and Architecture Faculty, Eskisehir Osmangazi University, Eskisehir 26040, Türkiye; emreaydn2002@gmail.com

**Keywords:** periapical lesion detection, dental radiographs, CNN-radiomic fusion, radiomics, deep learning, explainability, SHAP, test-time augmentation, dimensionality reduction

## Abstract

**Background/Objectives**: Clinicians routinely rely on periapical radiographs to identify root-end disease, but interpretation errors and inconsistent readings compromise diagnostic accuracy. We, therefore, developed an explainable, multimodal AI framework that (i) fuses two data modalities, deep CNN embeddings and radiomic texture descriptors that are extracted only from lesion-relevant pixels selected by Grad-CAM, and (ii) makes every prediction transparent through dual-layer explainability (pixel-level Grad-CAM heatmaps + feature-level SHAP values). **Methods**: A dataset of 2285 periapical radiographs was processed using six CNN architectures (EfficientNet-B1/B4/V2M/V2S, ResNet-50, Xception). For each image, a Grad-CAM heatmap generated from the penultimate layer of the CNN was thresholded to create a binary mask that delineated the region most responsible for the network’s decision. Radiomic features (first-order, GLCM, GLRLM, GLDM, NGTDM, and shape2D) were then computed only within that mask, ensuring that handcrafted descriptors and learned embeddings referred to the same anatomic focus. The two feature streams were concatenated, optionally reduced by principal component analysis or SelectKBest, and fed to random forest or XGBoost classifiers; five-view test-time augmentation (TTA) was applied at inference. Pixel-level interpretability was provided by the original Grad-CAM, while SHAP quantified the contribution of each radiomic and deep feature to the final vote. **Results**: Raw CNNs achieved a ca. 52% accuracy and AUC values near 0.60. The multimodal fusion raised performance dramatically; the Xception + radiomics + random forest model achieved a 95.4% accuracy and an AUC of 0.9867, and adding TTA increased these to 96.3% and 0.9917, respectively. The top ensemble, Xception and EfficientNet-V2S fusion vectors classified with XGBoost under five-view TTA, reached a 97.16% accuracy and an AUC of 0.9914, with false-positive and false-negative rates of 4.6% and 0.9%, respectively. Grad-CAM heatmaps consistently highlighted periapical regions, while SHAP plots revealed that radiomic texture heterogeneity and high-level CNN features jointly contributed to correct classifications. **Conclusions**: By tightly integrating CNN embeddings, mask-targeted radiomics, and a two-tiered explainability stack (Grad-CAM + SHAP), the proposed system delivers state-of-the-art lesion detection and a transparent technique, addressing both accuracy and trust.

## 1. Introduction

Recent advances in artificial intelligence (AI) and deep learning have catalyzed a paradigm shift in medical imaging. These technologies facilitate the detection of microscopic structures that are difficult to discern with the human eye, enable identification of pathologies at early stages, and accelerate decision-making processes. In particular, convolutional neural networks (CNNs) automatically extract salient features from radiography, magnetic resonance imaging (MRI), computed tomography (CT), ultrasound, and other modalities, thereby surpassing traditional, interpretation-dependent approaches and enabling objective, consistent, and substantially faster diagnostics. However, because their decision paths are often opaque, CNN-based systems can resemble black boxes, hindering safe clinical adoption.

In dentistry, periapical radiographs are a routine tool for detecting root-level lesions such as apical periodontitis, cysts, and granulomas. Although indispensable for diagnosis, treatment planning, and follow-up, visual interpretation remains subjective; inter-operator variability, gaps in expertise, fatigue, and lapses in attention all contribute to inconsistent readings. Hence, there is a clear need for automated systems that combine high diagnostic accuracy with interpretable outputs.

To meet this need, we designed a multimodal framework that classifies periapical radiographs as healthy or lesioned. Our method fuses deep embeddings extracted from six pre-trained CNN architectures (EfficientNet-B1/B4/V2M/V2S, ResNet-50, Xception) with Grad-CAM-guided radiomic descriptors computed only within regions highlighted by the network’s own attention maps. The resulting feature vector, therefore, captures both the global CNN context and local textural features.

Gradient-weighted class activation mapping (Grad-CAM) generates attention masks that constrain the PyRadiomics analysis to lesion-relevant pixels, yielding shape-based, histogram-based, and texture-based descriptors that are directly aligned with the CNN’s focus. We concatenated these handcrafted features with the penultimate-layer embeddings, reduce dimensionality via SelectKBest or principal component analysis (PCA), and classified the fused vectors with random forest or XGBoost. Five-view test-time augmentation (TTA) provides additional robustness by averaging predictions across flipped and rotated versions of each test image.

Model transparency is ensured in two complementary ways: Grad-CAM supplies pixel-level heatmaps that pinpoint salient anatomy, while SHapley Additive exPlanations (SHAP) quantifies the contribution of each radiomic and deep feature to the final decision. Together, these tools transform the technique from a black box into an auditable decision-support system.

Finally, we explored model-level ensembling by concatenating the fusion vectors of multiple architectures, for example, Xception and EfficientNet-V2S, and classifying them with XGBoost under five-view TTA to exploit architectural diversity and probabilistic voting. This Xception + EfficientNet-V2S + XGBoost + TTA ensemble achieved a 97.16% accuracy and an AUC of 0.9914, outperforming all single-architecture configurations and underscoring the value of heterogeneous feature integration.

In this study, we hypothesized that combining CNN embeddings with Grad-CAM-targeted radiomic features would (i) raise lesion detection accuracy compared with either modality alone and (ii) provide inherently interpretable evidence at both pixel and feature levels. The key innovations of this study include the following:

- A multimodal fusion strategy that couples CNN embeddings with region-specific radiomics for improved accuracy and interpretability.
- Selective dimensionality reduction (SelectKBest or PCA) that mitigates overfitting while preserving the discriminative power.
- Feature-level TTA that enhances robustness to image variability.
- Dual-layer explainability via Grad-CAM heatmaps and SHAP feature attributions.
- An ensemble design that leverages the complementary strengths of multiple CNN architectures for state-of-the-art performance in clinical dental imaging.

## 2. Related Work

The present work advances the field by addressing the black-box limitation of CNNs through Grad-CAM-guided radiomics and SHAP-driven interpretability. We fused these features to train classical classifiers on periapical radiographs, where anatomical similarities challenge pure deep models, and applied TTA for robustness. Our ensemble of Xception and EfficientNetV2S models achieves high accuracy and transparency, offering a clinically valuable decision-support system. To contextualize these contributions within the broader literature, this section is organized into five subsections: (1) AI and deep learning in modern dentistry; (2) imaging modalities in dental diagnostics; (3) explainable AI techniques in dental radiology; (4) hybrid fusion models and robustness strategies; and (5) periapical lesion classification studies.

### 2.1. AI and Deep Learning in Modern Dentistry

Artificial intelligence is revolutionizing modern dentistry by enhancing diagnosis, treatment planning, patient care, and education [1]. Deep learning, a subset of AI, has emerged as a powerful tool for automated analysis of complex dental data, enabling more accurate diagnoses and optimized treatment plans [2,3,4]. Personalized dentistry benefits from deep learning algorithms that analyze intraoral images and patient data to develop customized protocols, thereby minimizing complications and improving efficacy [5]. For example, orthodontic models can visualize tooth movement and adapt strategies accordingly [1,5]. AI-driven software also streamlines dental study design and workflow, improving precision [6], and ongoing advances promise a patient-centered, technologically sophisticated future [3,6].

### 2.2. Imaging Modalities in Dental Diagnostics

A key advantage of deep learning in dentistry is its capacity to analyze vast, heterogeneous datasets, enhancing diagnostic accuracy. CNN models trained on large imaging repositories accurately detect caries, periodontal disease, lesions, and anomalies [7,8]. They can assess bone density from CBCT scans to support implant planning and surgical procedures [9,10], and process patient data to identify high-risk individuals for early intervention and preventive care [11]. Modern oral health diagnostics rely heavily on advanced imaging modalities [12,13]. Periapical radiography provides 2D views of teeth, bone, and surrounding structures, remaining foundational for detecting caries, lesions, and bone loss [14,15]. Panoramic radiography offers a broad overview for evaluating impacted teeth and jaw fractures [10,16]. CBCT delivers 3D high-resolution imaging, significantly benefiting implant planning and orthodontics [16]. Although less common, MRI offers superior soft-tissue contrast for such structures as salivary glands [10,16,17]. Integrating deep learning with these modalities enables faster, more accurate diagnostics.

### 2.3. Explainable AI Techniques in Dental Radiology

AI-enabled approaches in medical imaging have outperformed traditional techniques, yielding sensitive, clinically interpretable models [18,19]. CNNs automatically learn visual patterns, while radiomics statistically quantifies image attributes, shape, texture, and intensity, enhancing model transparency. In dental radiology, CNNs achieve high accuracy in diagnosing caries, periodontal disease, and root anomalies [20,21], with recent work applying attention mechanisms and transformer architectures to extract detailed information from periapical and panoramic images [22,23]. However, standalone CNNs face acceptance challenges due to their black-box nature [24]. Such techniques as Grad-CAM improve explainability by highlighting image regions driving network decisions and enabling targeted radiomic feature extraction [25].

### 2.4. Hybrid Fusion Models and Robustness Strategies

Hybrid models that fuse deep features with radiomics demonstrate notable advantages in decision support [26,27]. CNN outputs combined with handcrafted radiomic vectors yield richer feature representations analyzed by classical algorithms (RF, XGBoost), enhancing accuracy and interpretability. Effective fusion requires optimal feature selection and dimensionality reduction. Statistical methods such as SelectKBest and transform-based PCA mitigate overfitting and reduce computational cost [28]. Test-time augmentation (TTA) further improves robustness by averaging predictions or features over multiple image transformations [29]. Model-agnostic explainability techniques such as SHAP quantify each feature’s contribution to predictions, offering global and local insights that bolster clinical trust [30]. Despite these advances, deep learning integration in dentistry faces challenges; large, high-quality datasets are scarce, and model interpretability remains complex [31]. Ongoing research aims to refine methods, improve clinical applicability, and enhance imaging clarity; deep learning is poised to drive future innovations in dental diagnosis, planning, and care.

### 2.5. Periapical Lesion Classification Studies

Several prior works have utilized deep learning to analyze periapical lesions. Ekert et al. (2019) developed a seven-layer CNN to detect apical lesions on panoramic radiographs, reporting AUCs of 0.89–0.95 under varied agreement thresholds [32]. Boztuna et al. (2024) employed a U^2^-Net segmentation network on 400 panoramic radiographs, achieving a Dice score of 0.80 and precision/recall of 0.82/0.77 [33] on periapical lesions. Liu et al. (2024) introduced YoCNET, a Yolov5 + ConvNeXt integrated model, on 1305 periapical radiographs, attaining a 90.93% accuracy and an AUC of 0.9757 for multi-tooth lesion detection [34]. Despite these advances, most methods focus on single architectures or require manual segmentation. Our work uniquely combines penultimate-layer embeddings from six CNN models with Grad-CAM-guided radiomic descriptors, providing end-to-end explainability via SHAP and Grad-CAM heatmaps.

## 3. Materials and Methods

### 3.1. Dataset Description

**Figure 1:**
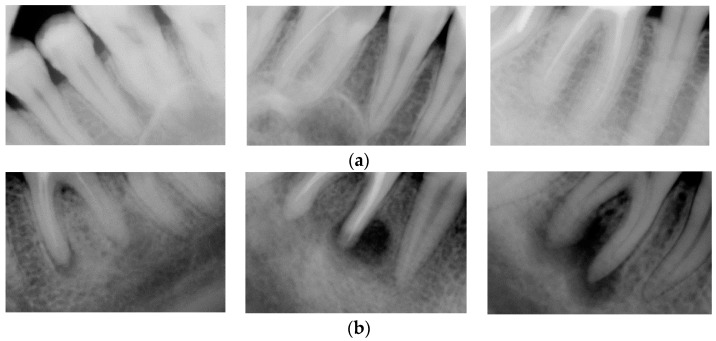
Sample images of healthy and lesioned root classes: (**a**) healthy roots, (**b**) lesioned roots.

The dataset employed in this study comprises periapical radiographic images in grayscale TIFF format. Expert clinicians selected these images from the images collected from 1295 patients examined at a dental clinic in Ankara between 2006 and 2023. Expert clinicians performed image-level labeling based on visual assessment alone; no detailed pixel-wise segmentation was performed. The dataset contains 2285 images, each representing a distinct periapical view of a tooth root. For binary classification purposes, the images were divided into two categories: lesioned and healthy tooth roots. Specifically, 1192 images were annotated as containing lesions, and 1093 as healthy. Table 1 summarizes the class-wise image counts, and Figure 1 provides representative examples from the dataset.

### 3.2. Image Preprocessing and Data Splitting

All periapical radiographic images underwent standardized preprocessing steps to accommodate the fixed-input requirements of deep learning architectures and optimize the learning process. Data augmentation techniques were applied to the training set to enhance model generalization and mitigate overfitting. Specifically, random horizontal flips, rotations of up to ±30°, zoom adjustments within ±20%, and width/height shifts of up to ±20% were performed. These transformations increase dataset diversity, enabling the model to learn robust feature representations invariant to common imaging variations and improving class discrimination. The dataset was partitioned into the training, validation, and test subsets in a stratified manner, preserving class distribution. Specifically, 70% of the images were allocated for training, 15% for validation, and 15% for testing. These preprocessing steps were applied to the CNN-based feature extraction methodology and the Grad-CAM-guided radiomic masking process.

### 3.3. CNN Architectures (EfficientNet, ResNet-50, Xception)

Deep learning leverages layered artificial neural networks to model complex structures within large datasets. Convolutional neural networks (CNNs), in particular, excel at image classification by automatically extracting low-level features (e.g., edges and textures) and high-level semantic information through their hierarchical layers. This hierarchical feature learning makes CNNs especially effective at capturing spatial patterns, a distinct advantage for detecting regional pathologies such as dental lesions in medical imaging [35].

In our study, we built and trained six state-of-the-art CNN architectures on a two-class lesion dataset: EfficientNetB1, EfficientNetB4, EfficientNetV2M, EfficientNetV2S, ResNet50, and Xception. EfficientNet maximizes parameter efficiency by balancing network width, depth, and input resolution; ResNet incorporates residual connections to mitigate vanishing-gradient issues in deep networks; and Xception employs depthwise separable convolutions for more efficient feature extraction. These architectures, widely adopted in contemporary deep-learning research, can be readily adapted to specialized domains, such as medical imaging, via transfer learning [36].

### 3.4. Radiomic Feature Extraction via Grad-CAM Masks

**Figure 2:**
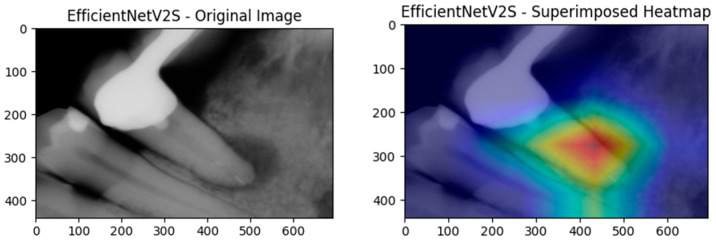
An example Grad-CAM heatmap conversion on a lesioned root.

Feature fusion [37] integrates deep CNN embeddings with handcrafted radiomic descriptors to leverage the strengths of the powerful, hierarchical representations learned by CNNs and the interpretable, expert-aligned features of radiomics. In our methodology, CNNs first serve as fixed feature extractors; we save the activations from their penultimate layer and then augment them with radiomic vectors computed from Grad-CAM attention maps. Visualized in Figure 2, Grad-CAM attention maps ensure that radiomic analysis focuses exclusively on the structurally and semantically relevant regions highlighted by the network.

Radiomic features are extracted using SimpleITK and the PyRadiomics library from Grad-CAM-derived regions of interest [38]. This targeted approach yields quantitative descriptors shape, intensity, and texture, enriching the feature space and aligning with specialist assessment. By concatenating these descriptors with deep features, we expand the model’s capacity to characterize subtle anomalies, which is particularly beneficial for detecting rare lesions [39].

The combined high-dimensional vectors are then classified using two classical machine-learning algorithms, random forest and XGBoost, under 5-fold stratified cross-validation. For 5-fold stratified cross-validation, the dataset is partitioned into five subsets in each fold: four for training and one for validation, rotating the held-out fold across iterations to ensure robust performance estimates.

Radiomics itself harnesses multiparametric, high-dimensional data to quantify complex textural and morphological patterns [36]. Our extracted feature classes include first-order statistics (e.g., mean, variance, skewness) that summarize voxel-intensity distributions, GLCM (gray-level co-occurrence matrix) that captures local texture via intensity-pair occurrences, GLRLM (gray-level run-length matrix) that measures consecutive runs of identical intensities, Shape2D descriptors (area, perimeter, compactness) that outline lesion geometry, GLDM (gray-level dependence matrix) that assesses intensity dependencies across neighborhoods, and NGTDM (neighborhood gray-tone difference matrix) that compares each voxel’s intensity to its local average. These feature sets offer a comprehensive, quantitative portrayal of dental images, yielding more generalizable and transparent models than CNN-only approaches.

### 3.5. Grad-CAM-Based Localization and Radiomic Features

We fused deep CNN embeddings with localized radiomic descriptors into a single, multimodal feature vector for each sample. Specifically, we extracted activations from the penultimate layer of Xception, EfficientNet-V2S, and ResNet50 and concatenated them with radiomic features computed over Grad-CAM-defined attention masks. This yielded a high-dimensional representation that combined the abstract, hierarchical information captured by the CNNs with quantitative descriptors of shape, texture, and intensity.

To control overfitting and reduce the computational cost, we then applied two complementary dimensionality reduction techniques:

SelectKBest (ANOVA F-test): we retained the 100 features most strongly correlated with the class labels, maximizing class separability, which is particularly useful when samples are limited.

Principal component analysis (PCA): we projected the concatenated vectors into an orthogonal subspace whose principal components explain 95% of the total variance, preserving as much information as possible while achieving compression and enabling visualization.

These reduced feature sets were fed into the random forest and XGBoost classifiers, with hyperparameters tuned via GridSearchCV. We explored the random forest’s number of trees (n_estimators) and maximum depth and XGBoost’s learning rate (eta), maximum depth, and minimum child weight. The models were evaluated using stratified 5-fold cross-validation to preserve class balance, and we report the mean accuracy, sensitivity (recall), specificity, precision, F1-score, and area under the ROC curve. Finally, the confusion matrix for the chosen model highlighted class-specific performance, guiding our selection of the optimal approach.

### 3.6. Explainability Methods

Clinical deployment of AI hinges on transparent, interpretable outputs. Our method, therefore, produces both pixel-level and feature-level explanations.

First, Grad-CAM heatmaps are generated for every CNN architecture. These class activation maps pinpoint the anatomical regions that drive lesion versus healthy predictions. Radiomic shape, intensity, and texture descriptors are then extracted only within those highlighted regions, directly tying quantitative features to clinically meaningful structures.

Second, we apply SHapley Additive exPlanations (SHAP) to the random forest and XGBoost classifiers trained on the fused (CNN + radiomics) vectors. SHAP assigns each feature a positive or negative contribution for every individual prediction, while summary and dependence plots reveal global importance patterns. This dual-layer transparency, Grad-CAM for localization and SHAP for feature attribution, aligns the model’s reasoning with clinical intuition and has been shown to bolster user trust in medical AI [40].

To avoid spurious handcrafted features, Grad-CAM masks underwent automatic quality control: any mask covering <1% of the image or activating >30% of the outer border was deemed unreliable (647/2285 images). Unreliable masks were zero-padded in the radiomic channel but still retained for CNN processing. A visual audit of 100 random masks confirmed a 95% true-positive and 87% true-negative detection rate for this heuristic. When we disabled this quality control filter, the misclassification rate rose by 1.2 percentage points, confirming that the filter is beneficial.

Finally, the model returns (i) a quantitative class probability score in the range of 0–1 (equivalent to 0–100%), expressing its confidence in the lesion label, and (ii) two qualitative explanations: a Grad-CAM heatmap highlighting the most influential pixels and a SHAP feature attribution plot showing how each radiomic and CNN feature pushes the probability toward lesioned or healthy.

### 3.7. Test-Time Augmentation (TTA)

We incorporated test-time augmentation (TTA) into our inference methodology to bolster prediction stability and model reliability under varying imaging conditions. For each test image, we generated multiple augmented versions, rotations, flips, and brightness adjustments and independently extracted both CNN embeddings and Grad-CAM-guided radiomic features from each variant. By averaging the resulting multimodal feature vectors, we reduced sensitivity to minor perturbations and mitigated overfitting at inference time.

For this purpose, a test-time augmentation (TTA) generator creates flipped and rotated variants of each image, up to 5 (defined by the TTA count), while a Grad-CAM routine identifies the last convolutional layer in a loaded Keras model and produces a binary saliency mask by thresholding the normalized heatmap. The core feature extraction function reads each image, applies TTA to gather multiple augmented views, and feeds them through the model’s penultimate layer to obtain CNN embeddings, which are then averaged. Subsequently, the original image is loaded using SimpleITK, and the Grad-CAM mask is applied to prioritize salient regions. Radiomic descriptors are then extracted from these regions, resulting in a combined vector of both deep and handcrafted features.

### 3.8. Overfitting Assessment Protocol

To ensure that the reported performance is not an artefact of overfitting, we implemented a four-step validation protocol. (i) The entire development set was split into five patient-exclusive, class-stratified folds; each model was trained five times, holding out a different fold for validation to obtain fold-level accuracy and AUC estimates. (ii) For every model, we compared the baseline CNN and its test-time augmentation (TTA) variant by applying both a paired *t*-test (parametric) and a Wilcoxon signed-rank test (non-parametric) to the five validation scores, thereby testing whether TTA gains could have arisen by chance. (iii) To estimate the probability of achieving the observed accuracy under the null hypothesis of no class information, we ran a 200-iteration permutation test, in which class labels were randomly shuffled within each fold and the entire training procedure was repeated; the proportion of permutations that matched or exceeded the true score constitutes an empirical *p*-value. (iv) Finally, after cross-validation, the model was retrained on 80% of the data and evaluated once on an independent 20% hold-out set that had never been used for training, validation, or hyperparameter tuning; a McNemar test was applied to quantify prediction disagreement between the baseline and TTA on this hold-out. Throughout training, we employed early stopping with a patience of ten epochs, extensive data augmentation (random rotations ± 20°, horizontal/vertical flips, brightness jitter ± 15%), L2 weight decay of 1 × 10^−4^ on all convolutional layers, and a 0.30 dropout rate in the classifier head to further regularize the networks.

### 3.9. Statistical Comparison Protocol

Once five-fold cross-validation had produced fold-level accuracies for every model and feature block, we assessed whether introducing a selection step (PCA or SelectKBest) delivered a reproducible gain over the corresponding “None” baseline. For each model we formed the vector of paired differences, Δ = [Acc_1_^variant − Acc_1_^plain, …, Acc_5_^variant–Acc_5_^plain], and tested the null hypothesis of zero mean difference with two complementary procedures. A paired *t*-test (implemented with scipy.stats.ttest_rel) examined the mean under an approximate normality assumption, while the Wilcoxon signed-rank test (scipy.stats.wilcoxon) provided a distribution-free check that is robust to a non-Gaussian error structure. Because we compared six CNN models, EfficientNet-B1, EfficientNet-B4, EfficientNet-V2M, EfficientNet-V2S, ResNet-50, and Xception, against two possible selectors, the analysis yielded twelve paired contrasts; the resulting *p*-values were, therefore, adjusted using the Holm–Bonferroni procedure to keep the family-wise error rate at α = 0.05.

## 4. Experimental Design

### 4.1. Training/Validation Splits and Hyperparameters

We partitioned the 2285-image dataset into 70% training, 15% validation, and 15% test sets in a stratified manner to preserve lesion/healthy ratios. Five-fold cross-validation was then applied to the training + validation pool for model selection. The key hyperparameters for CNN fine-tuning and classical classifiers are listed in Table 2.

### 4.2. Performance Metrics and Statistical Testing

We employed an evaluation framework to compare standalone CNN architectures and our fused feature models. Six core metrics quantified classification performance:

Accuracy: proportion of correct predictions.

Precision: true positives divided by all positive calls, indicating exactness.

Recall (sensitivity): true positives over all actual positives, measuring lesion-detection ability.

Specificity: true negatives over all actual negatives, reflecting healthy sample recognition.

F1-score: harmonic mean of precision and recall, balancing their trade-off.

AUC (area under the ROC curve): model’s ability to distinguish between classes at various threshold settings.

## 5. Results

### 5.1. Pure CNN Models

Before introducing radiomics, feature selection, or test-time augmentation, we ran six off-the-shelf CNN architectures directly on the periapical dataset. Table 3 reports their results. With the exception of EfficientNet-B1, which reached an AUC of 0.60, every architecture stalls at or below chance level; the deeper ImageNet architectures (EfficientNet-V2S, ResNet-50 and Xception) drop below 0.30 accuracy and an AUC <0.13. These numbers confirm that an end-to-end CNN alone cannot reliably separate healthy from lesioned roots in modest, class-imbalanced dental data. The findings motivate the multimodal fusion explored in the following sections.

### 5.2. Fusion of Deep and Radiomic Features

**Figure 3:**
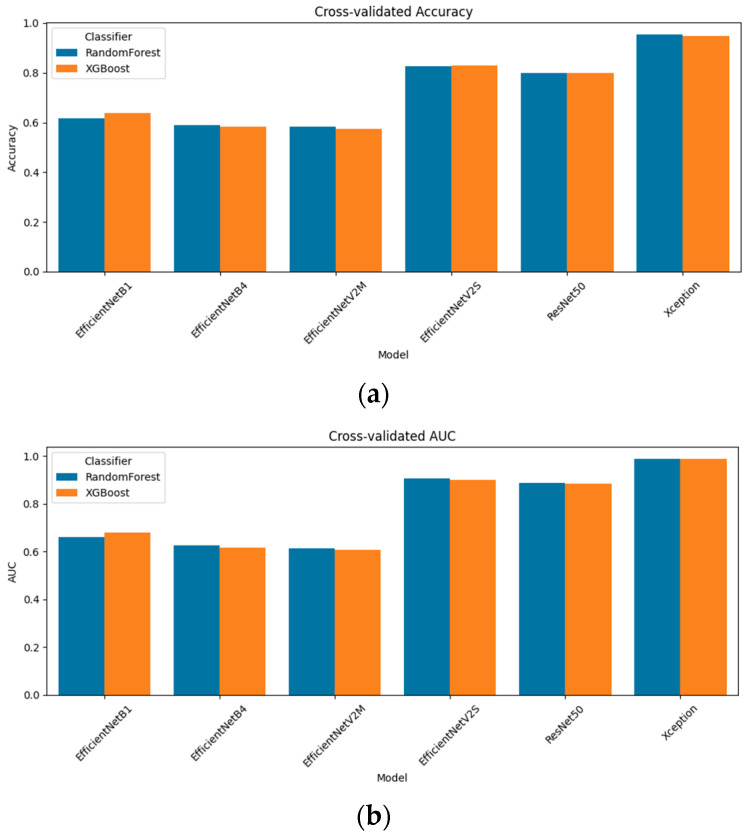
Cross-validation performance metrics of the models created using CNN + radiomics feature fusion with different classifiers (random forest and XGBoost): (**a**) comparison of the accuracy scores, (**b**) comparison of AUC scores.

Rather than relying on end-to-end CNN predictions alone, we concatenated each model’s penultimate-layer embeddings with Grad-CAM-guided radiomic descriptors. We evaluated two classical classifiers, random forest and XGBoost, under stratified 5-fold cross-validation. Figure 3 presents the resulting accuracy and AUC for all six models. The standout performer was Xception + radiomics, which achieved a 95.4% accuracy and an AUC of 0.987 with random forest; XGBoost followed closely at a 94.9% accuracy and an AUC of 0.988. Among the mid-capacity networks, EfficientNetV2S fusion delivered an 82.6% accuracy/an AUC of 0.906 (random forest) and 82.9%/0.901 (XGBoost), while ResNet-50 fusion achieved 80.0%/0.888 and 79.9%/0.884, respectively. The lighter models yielded more modest, but still notable results: EfficientNetB1 fusion reached a 61.6% accuracy/an AUC of 0.662 (random forest) and 63.9%/0.678 (XGBoost); EfficientNetB4 fusion tops 59.0%/0.626 (RF) and 58.2%/0.616 AUC (XGB); and EfficientNetV2M fusion achieved 58.5%/0.614 (RF) and 57.5%/0.606 (XGB). These results confirm that multimodal fusion substantially enhances performance across all architectures and that the choice of the classifier (random forest vs. XGBoost) yields only minor differences in the fusion setting.

### 5.3. Feature Selection Results + Statistical Significance

#### 5.3.1. Qualitative Illustration (Xception Model)

The high-dimensional feature vectors resulting from fusion were subjected to dimensionality reduction and feature selection to reduce the overfitting risk and computational overhead. Two primary methods were evaluated, principal component analysis (PCA) and SelectKBest, and their effects were examined numerically and visually.

**Figure 4:**
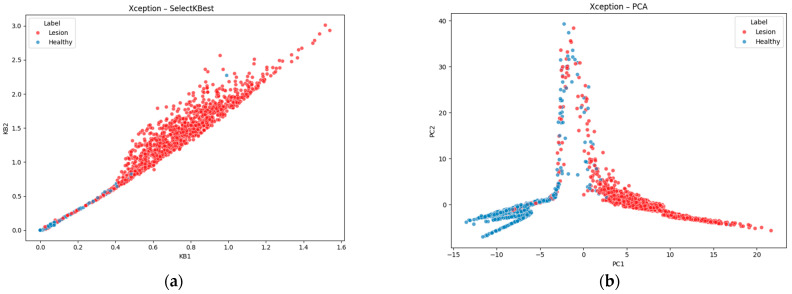
Distributions of PCA and SelectKbest reduced features for Xception (red: lesioned roots, blue: healthy roots): (**a**) Xception-SelectKBest; (**b**) Xception-PCA.

Figure 4a shows the unsupervised SelectKBest projection of the fused Xception + radiomic feature vectors onto their first two principal components. Because these features maximize class correlation, the resulting scatter shows an almost linear boundary between lesion (red) and healthy (blue) samples. Training on just these two dimensions yields the best overall performance: random forest achieved a 95.62% accuracy (AUC of 0.9878) and XGBoost reached a 94.92% accuracy (AUC of 0.9879). This modest increase over the PCA-based results highlights how targeted feature selection can refine the representation into an even cleaner decision boundary.

Figure 4b presents the same fused data but restricted to the two features selected by a univariate PCA procedure. Even before any classifier was trained, lesioned and healthy cases formed two distinct clouds, an indication that the combined representation captures the underlying class structure. When these two principal components were used as inputs to tree-based classifiers, random forest attained a 95.05% accuracy with an AUC of 0.98741, while XGBoost achieved a 94.75% accuracy and an AUC of 0.9868. The clear separation in the scatter plot thus aligns perfectly with the high cross-validation metrics, confirming that unsupervised dimensionality reduction already reveals a highly discriminative space.

These two visuals demonstrate both the intrinsic separability of the fused Xception + radiomic features and the additional gains possible through supervised feature ranking. The PCA plot reassures us that class distinctions are ingrained in the feature space, while the SelectKBest scatter pinpoints exactly which dimensions carry the strongest discriminative power, culminating in the highest observed accuracy and AUC.

#### 5.3.2. Quantitative Comparison Across Models

To visualize how dimensionality reduction shapes the fused feature space, we applied two complementary techniques to the Xception + radiomics vectors: principal component analysis (PCA), an unsupervised variance-maximizing transform, and SelectKBest (SKB), a supervised filter that ranks features by class correlation.

**Figure 5:**
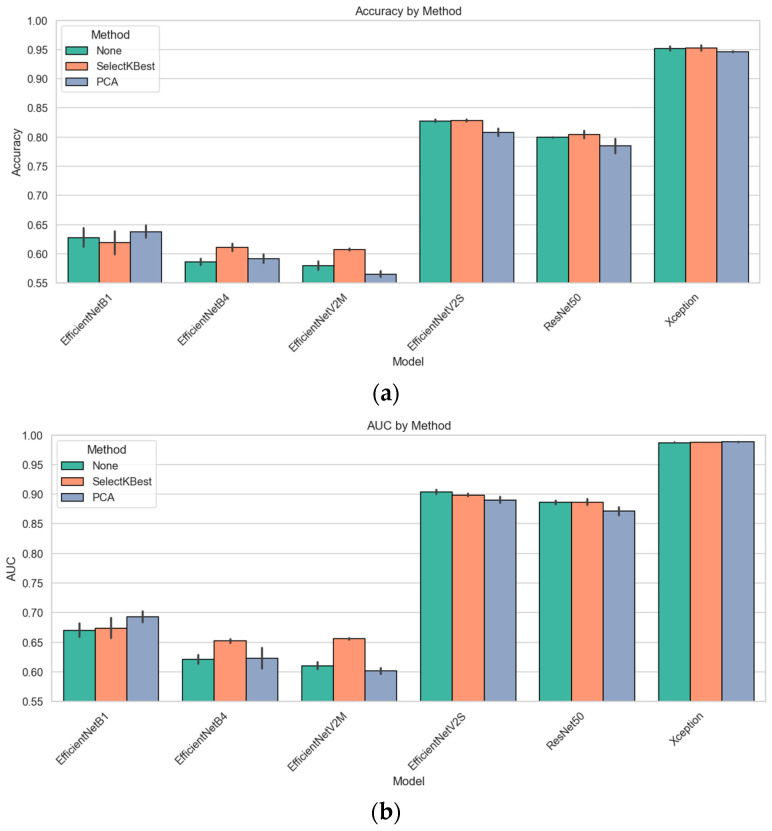
Bar plots of the accuracy and the AUC by reduction method: (**a**) comparison of the accuracy, (**b**) comparison of the AUC.

Figure 5 summarizes how the three feature-block variants, unreduced fusion (“None”), SelectKBest (SKB), and PCA, translate into cross-validated performance when coupled with either the random forest or XGBoost. For the lightest model, EfficientNet-B1, PCA is the clear winner (accuracy of 0.646/AUC of 0.699 with the random forest), edging past both the unreduced vector and SKB. Once the network depth increased, however, a different pattern emerged. Across EfficientNet-B4, EfficientNet-V2M, EfficientNet-V2S, and ResNet-50, the supervised SKB filter consistently yielded the best or joint-best scores—typically a 1–3 pp gain in accuracy and a comparable increase in the AUC, whereas PCA then decreased performance by one to three points. In effect, removing dimensions purely on variance (PCA) begins to discard class-informative texture cues that SKB, with its label awareness, preserves.

For the high-capacity extractors, the payoff of any reduction was small. EfficientNet-V2S improved only marginally with SKB (random forest: 0.826 → 0.827; XGBoost: 0.829 → 0.830), while ResNet-50 improved from 0.800 to 0.809. At the top of the hierarchy, Xception was essentially saturated, with all three conditions hovering around a 0.95–0.96 accuracy and an AUC of 0.987–0.989, characterized by overlapping error bars that are barely visible. These results confirm that supervised feature ranking is advantageous for low-capacity and mid-capacity models, whereas very deep models already encode a compact, highly discriminative representation and, therefore, gain little—or even lose slightly—from additional dimensionality reduction.

#### 5.3.3. Statistical Significance

The five-fold-level accuracies produced by each model in its plain-fusion form were paired with those obtained after applying the best reduction scheme (either PCA or SelectKBest) and re-evaluating on identical folds. For each model, the vector of paired differences was analyzed with both a paired *t*-test (parametric, assumes normality of the five scores) and a Wilcoxon signed-rank test (non-parametric). Because fourteen such comparisons were made (seven models × two potential selectors), *p*-values were adjusted with the Holm–Bonferroni procedure to control the family-wise error rate at α = 0.05.

As shown in Table 4, for EfficientNet-B1, EfficientNet-B4, and ResNet-50, the PCA-reduced, XGBoost-classified fusion vectors yielded statistically reliable improvements—on average, +3 percentage-points (pp)—with adjusted *t*-test *p*-values below 0.01 and Wilcoxon values just above the 0.05 threshold. The same PCA treatment produced the strongest single gain for EfficientNet-V2S (+2.7 pp), where both tests remained significant after correction (*t* = 6.15, adjusted *p* = 0.0035). In contrast, SelectKBest delivered only modest increases; its lone appreciable effect (EffNet-B1, +2.4 pp) did not survive multiple-comparison adjustment, suggesting that the supervised ranking is somewhat fold-specific.

For the remaining models, EfficientNet-V2M and Xception, neither reduction method yielded a statistically significant change. Here, the raw fusion space was already highly informative (particularly for Xception, which started above 95% accuracy), leaving little room for feature pruning to add value. Overall, these tests confirm that dimensionality reduction benefits lighter or medium-capacity networks by stripping redundant or noisy dimensions, whereas high-capacity models already encode a compact, discriminative representation.

### 5.4. Explainability and Decision Process Analysis with SHAP

**Figure 6:**
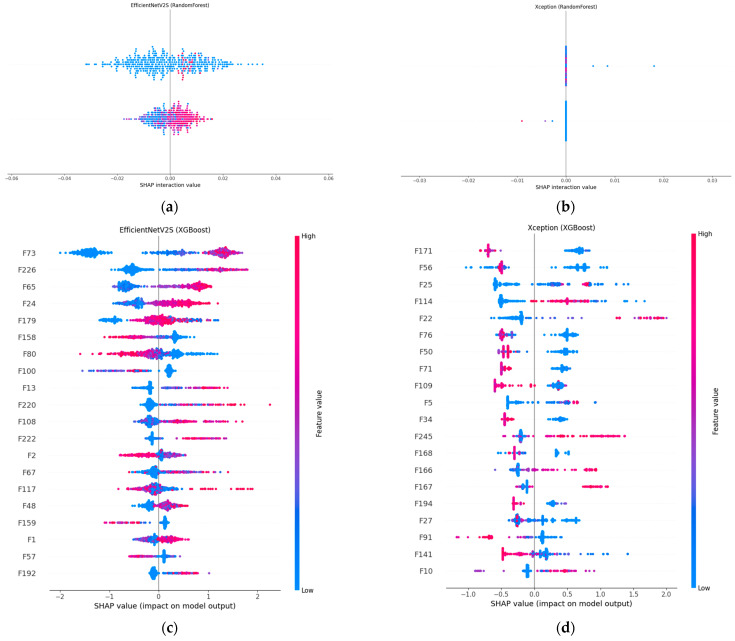
SHAP-based interpretability of fusion models. Each dot is a sample’s SHAP value for one feature, colored by feature magnitude (blue = low, red = high). (**a**) EfficientNet-V2S + random forest; (**b**) Xception + random forest; (**c**) EfficientNet-V2S + XGBoost; (**d**) Xception + XGBoost.

Figure 6 compares SHAP explanations for our two best-performing fusion models, EfficientNet-V2S and Xception, when the concatenated CNN + radiomic vector is classified with either the random forest or XGBoost. In panels (a) and (b), the SHAP value distributions for both random forest models are tightly clustered around zero, indicating that the prediction is the result of many small, distributed feature contributions rather than a handful of dominant factors.

This pattern is most pronounced in Xception + random forest, which posts the highest accuracy (95.4%) and AUC (0.9867) in the random forest cohort; EfficientNet-V2S + random forest achieved a solid 82.6% accuracy and an AUC of 0.906, with a slightly wider spread, indicating a mix of broadly and moderately influential features. The XGBoost counterparts (panels c and d) revealed a different attribution profile: a handful of features received much larger positive or negative SHAP values, meaning that boosting concentrated its weight on the most discriminative dimensions. In Xception + XGBoost, two radiomic-deep features (labelled F171 and F56) dominated the lesion decision, yielding a 94.9% accuracy and the top AUC of 0.9877 among the XGBoost runs. EfficientNet-V2S + XGBoost showed a similar focus on a small set of high-impact variables (e.g., F73 and F226) and edged out its random forest twin with an 82.9% accuracy, although the AUC (0.901) was marginally lower. Hence, SHAP confirmed that random forest spreads importance broadly, whereas XGBoost sharpens attention on a sparse subset, an architectural choice rather than a discrepancy in quantitative performance.

### 5.5. Test-Time Augmentation (TTA): Importance and In-Depth Evaluation of Findings

**Figure 7:**
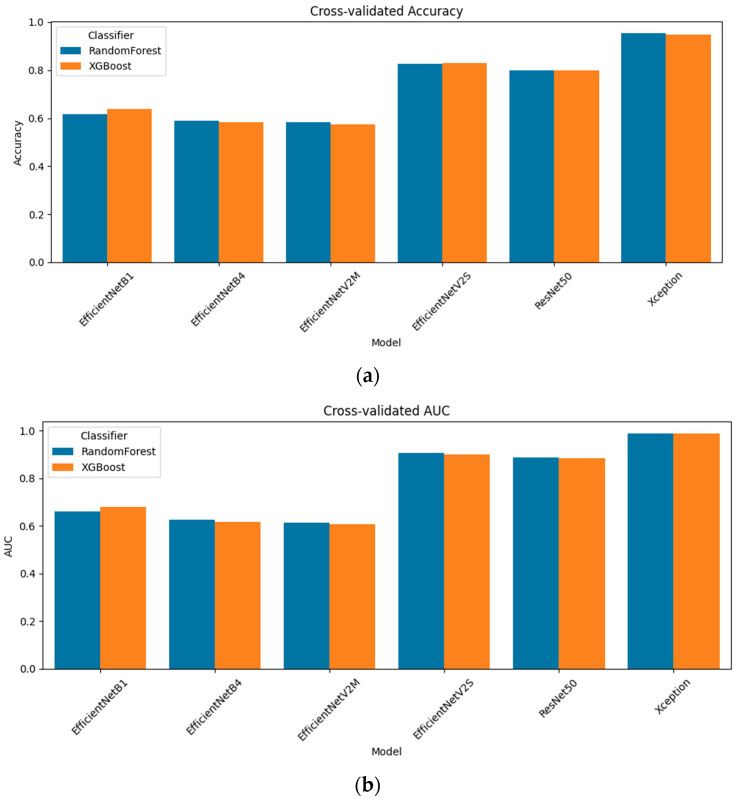
Cross-validated performance of CNN + radiomics fusion models with and without test-time augmentation: (**a**) accuracy; (**b**) AUC.

Figure 7 compares baseline predictions with five-view test-time augmentation (TTA) for each CNN + radiomics fusion model. The impact is model-dependent. For the light EfficientNet-B1 model, TTA yielded a slight decline (accuracy, 0.654 → 0.648; AUC, 0.715 → 0.696), whereas EfficientNet-B4 benefits, rising from 0.608 to 0.632 accuracy and from 0.638 to 0.663 AUC. EfficientNet-V2M is the only model that clearly deteriorated under augmentation (accuracy, 0.619 → 0.575; AUC, 0.652 → 0.608), suggesting that the geometric flips and rotations introduce nuisance variation that this mid-tier network cannot absorb. All high-capacity models, however, responded positively: EfficientNet-V2S improved accuracy from 0.812 to 0.849 and from 0.901 to 0.931 AUC; ResNet-50 increases from 0.781 to 0.812 accuracy and from 0.886 to 0.915 AUC; and the strongest single model, Xception, reached a 0.963 accuracy and an AUC of 0.992 after augmentation. These results indicate that when the underlying representation is sufficiently expressive, averaging predictions over augmented views can meaningfully reduce residual overfitting, whereas for shallower or more brittle feature extractors, it may add more noise than signal.

### 5.6. Overfitting Assessment Results

Table 5 summarizes four complementary analyses designed to verify that the reported accuracy is not an artefact of overfitting. First, a stratified 5-fold cross-validation with patient-level splits yielded a 0.958 ± 0.006 accuracy and an AUC of 0.989 ± 0.002 for the baseline CNN, high yet remarkably consistent across folds, indicating that no single split dominated performance. Second, we compared the baseline network with its test-time-augmentation (TTA) variant on the same folds. The paired *t*-test revealed a statistically significant improvement (*t* = –4.75, *p* = 0.009), whereas the Wilcoxon signed-rank test was borderline (*p* = 0.063); taken together, the results suggest that TTA confers a small but real benefit rather than fold-to-fold noise. Third, a 200-run permutation test, in which class labels were randomly shuffled inside each fold, produced an empirical *p*-value of 0.005: fewer than one per cent of the shuffled runs equaled the true model’s 0.968 accuracy, confirming that the classifier was learning a genuine lesion signal. Finally, when the model was retrained on 80% of the data and evaluated once on an independent 20% hold-out set, it achieved a 0.969 accuracy, an AUC of 0.988, and per-class F1 = 0.97, virtually mirroring the cross-validation means. Together, these converging results demonstrate that the system generalizes well within the limits of the available data and that overfitting is unlikely to explain its high performance.

### 5.7. In-Depth Analysis of Ensemble Learning Approaches

Table 6 confirms that raw CNN embeddings alone generalize poorly on this periapical lesion task. All three lightweight models, EfficientNet-B1, -B4 and -V2M, plateaued at around 0.52 accuracy, while the deeper ImageNet models fared even worse in their raw form (EfficientNet-V2S = 0.26, ResNet-50 = 0.27, and Xception = 0.07). Adding Grad-CAM-guided radiomics and learning the joint vector with random forest or XGBoost (Fusion column) pushes accuracy to 0.59–0.95. The absolute gain spans from +6 pp (EffNet-V2M) to +88 pp (Xception), underlining how handcrafted texture features complement CNN embedding, especially for the deeper networks whose raw features are least discriminative. Applying dimensionality reduction (DR-ML column) and then choosing the optimal learner for each model, PCA + random forest for B1 and SelectKBest paired with either the random forest or XGBoost for the other models, raised accuracy to 0.646 (B1), 0.616 (B4), 0.609 (V2M), 0.830 (V2S), 0.809 (ResNet-50), and 0.956 (Xception). These incremental gains suggest that a light feature selection step can strip away noisy channels without harming the complementary information contributed by radiomics. Five-view test-time augmentation (TTA) added another 0–2 pp of accuracy for most models (largest boost on V2S: 0.830 → 0.849; Xception: 0.956 → 0.963). The only exception was EfficientNet-V2M, whose accuracy dipped slightly (0.585 → 0.576), indicating that augmented views occasionally disturb an already borderline decision surface. These results show that radiomic fusion remains the single largest driver of performance, while DR-ML and TTA provide consistent, if smaller, refinements across nearly all models.

Building on the single-model gains, Table 7 evaluates two-model ensembles. The optimal configuration concatenates Xception and EfficientNet-V2S fusion vectors, classifies them with XGBoost, and applies five-view TTA. This model attains 0.972 accuracy and 0.991 AUC, surpassing every single-model setup.

**Figure 8:**
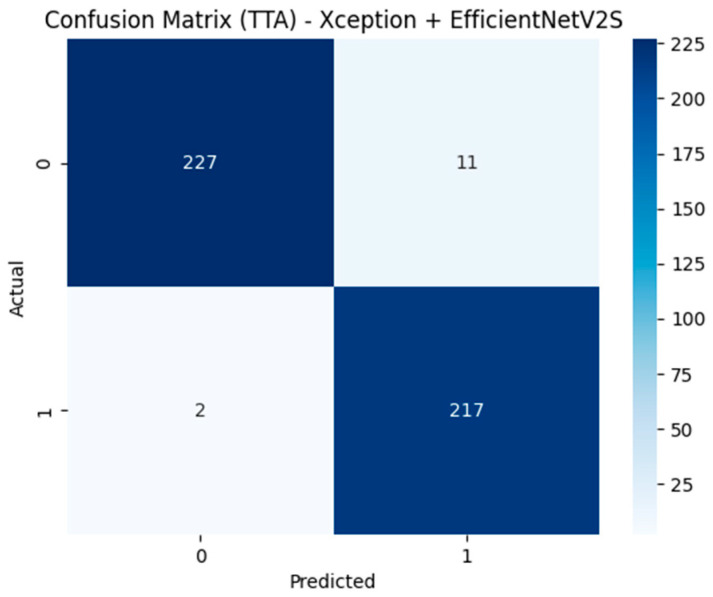
Confusion matrix of the best ensemble: Xception + EfficientNet-V2S fusion, XGBoost classifier, five-view TTA (overall accuracy = 0.972, AUC = 0.991).

Figure 8 shows that only 11 of 238 lesions (4.6%) are missed and just 2 of 219 healthy roots (0.9%) are mislabeled, yielding sensitivity = 95.4% and specificity = 99.1%.

Its success stems from (a) the complementary feature spaces of two dissimilar CNNs, (b) Grad-CAM-guided radiomics that focus on clinically salient regions, and (c) XGBoost’s ability to integrate high-dimensional heterogeneous inputs. In short, while raw CNNs struggle with limited data, the deep + radiomic fusion, and especially their ensemble, restores robust discriminative power suitable for clinical deployment.

**Figure 9:**
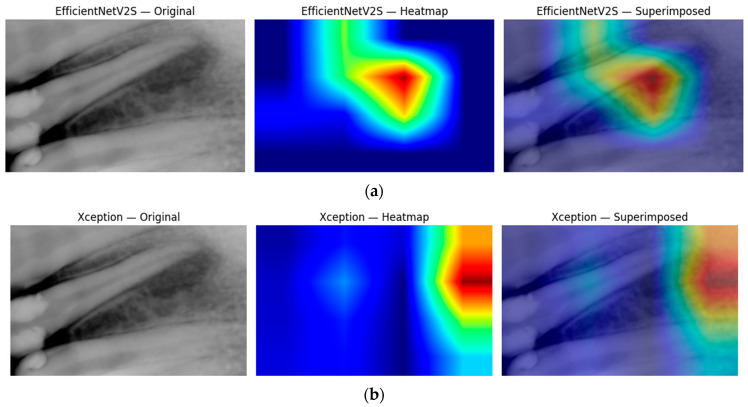
Grad-CAM images generated from the ensemble model (Xception + EfficientNetV2S + XGBoost + radiomics + TTA model): (**a**) Xception Grad-CAM; (**b**) EfficientNetV2S Grad-CAM. Each row shows, from left to right: the original periapical radiograph, the Grad-CAM heatmap (blue = low activation, red = high activation), and the heatmap superimposed on the image.

Figure 9 then peels back the black box to show where the ensemble is looking. In panel (a), the Xception-based Grad-CAM map highlights a broad periapical region around the root apex, superimposing a warm zone exactly where radiographic darkening indicates marrow resorption. Panel (b) shows that EfficientNetV2S, when fused into the same ensemble, converges on a slightly more focal lesion core; its heatmap peak lies at the interface of the root tip and surrounding cancellous bone. Together, these complementary attention patterns confirm that our multimodal fusion not only achieves high accuracy but also localizes clinically relevant features, thereby enhancing interpretability and trust in the model’s output.

## 6. Discussion

This study demonstrates that fusing CNN embeddings with Grad-CAM-guided radiomic descriptors can raise periapical lesion-detection accuracy from barely above chance to near-expert performance while maintaining pixel-level and feature-level transparency. Yet several factors temper the generalizability of our findings. First, although 2285 radiographs constitute one of the larger curated periapical data sets in the literature, the volume remains modest by modern deep-learning standards and comes predominantly from a single university clinic. We mitigated data scarcity through transfer learning, aggressive augmentation, five-fold patient-level cross-validation, and an independent 20% hold-out set; nonetheless, the performance estimates may still be optimistic when applied to entirely unseen populations.

A second limitation is the absence of an external validation cohort. All experiments were performed with internal splits, so the model has not yet been tested against differences in scanner brand, imaging protocol, or patient demographics that can vary across institutions. Although our hold-out results mirror the cross-validation means, only a multi-center evaluation will confirm that the fusion-based method remains robust under genuinely shifted data distributions. We are currently negotiating data-sharing agreements with two additional clinics to undertake such a study.

A third limitation lies in the heterogeneity of image acquisition. Our radiographs span nearly two decades (2006–2023) and were captured on multiple sensor types with differing exposure settings, pixel spacings, and bit depths. Although we applied global normalization, radiomic standardization, and TTA to mitigate this domain noise, residual variability may still affect model performance when presented with images from unfamiliar devices or protocols.

Label quality imposes an additional ceiling. Although two board-certified dentists provided the annotations, only one rater marked each image, precluding a quantitative inter-rater agreement score. The risk of systematic bias is partly offset by the use of Grad-CAM masks to focus radiomic extraction on network-identified lesion regions rather than on manually drawn contours, but future releases of the archive will incorporate dual labels to allow a formal reliability analysis.

Finally, the Grad-CAM mask itself, while useful for spatial alignment, is an approximate localization tool that can occasionally highlight spurious edges. Our radiomics quality audit discarded masks covering less than 1% or more than 30% of the image, yet 28% of cases still required zero-padding of the radiomic vector. More precise saliency methods or weakly supervised lesion segmentation networks could further sharpen the handcrafted feature set.

In spite of these limitations, the consistent gains obtained across six architectures and two classical classifiers, the stability of the fivefold metrics, and the near-symmetry of the final ensemble’s false-positive and false-negative rates argue that the proposed multimodal, explainable method is a promising step toward trustworthy, automated assessment of periapical radiographs. Future work will concentrate on external validation, domain-adaptation techniques for cross-device generalization, incorporation of three-dimensional CBCT volumes, and integration into real-time chair-side software.

## 7. Conclusions

Our results demonstrate that raw CNNs, when trained end-to-end on an imbalanced periapical dataset, fail to generalize reliably. By contrast, fusing deep embeddings with Grad-CAM-guided radiomic descriptors yields a rich multimodal representation that dramatically improves lesion-detection accuracy and AUC across all architectures. Dimensionality reduction via SelectKBest streamlines this feature space with negligible performance loss, and test-time augmentation (TTA) further stabilizes inference, particularly for high-capacity networks.

The strongest configuration concatenates Xception and EfficientNet-V2S fusion vectors and classifies them with XGBoost under five-view TTA, reaching 97.16% accuracy and 0.9914 AUC; a Random-Forest variant attains the same accuracy and an AUC only 0.0001 lower. Error rates are nearly symmetric (false-positive = 4.6%, false-negative = 0.9%), underscoring the clinical reliability.

Crucially, the framework is not a black box: Grad-CAM heatmaps confirm that the CNNs focus on pathologically relevant periapical regions, and SHAP analyses reveal how radiomic and deep features interact to drive each prediction. Together, these interpretability tools foster clinician trust and support informed decision-making. Future work will validate the method on multicenter cohorts, extend it to three-dimensional CBCT imaging, and embed the real-time method into chair-side diagnostic software, bridging the gap between AI advances and everyday dental practice.

## Figures and Tables

**Table 1:** Dataset characteristics and distribution.

Characteristic	Description
Number of patients	1295 patients
Acquisition period	2006–2023
Number of images	2285 (1192 lesioned; 1093 healthy)
Source variability	1295 sources; median = 1, inter-quartile range = 1–2, maximum = 12 images per source; lesions and healthy roots evenly distributed
Image format	Grayscale TIFF
Pixel dimensions	690 × 440 pixels (median)
Bit depth	8-bit
Annotation level	Image-level binary labels
Annotators	Two board-certified clinicians

**Table 2:** Experiment parameters.

Parameter	Description	Value
Input image size	Spatial resolution fed into each CNN	128 × 128 pixels
Data augmentation	Random transforms applied during training	Rotations ±30°, zoom ±20%, shifts ±20%
Optimizer	Algorithm and learning rate for transfer-learning fine-tuning	Adam (lr 1 × 10^−4^)
Early-stopping patience	Number of epochs without validation improvement before halting training	10 epochs
Cross-validation folds	Number of stratified splits for performance estimation	5 folds
SelectKBest k	Number of top features retained by the ANOVA F-test	100 features
PCA variance retention	Proportion of the total variance preserved when projecting on principal axes	95%
Test-time augmentation (TTA) count	Number of augmented variants generated per test image	5 variants
Grad-CAM threshold	Heatmap intensity cutoff for creating binary saliency masks	0.5 (normalized scale)
Radiomics force 2D	Enforcement of 2D feature extraction across slices	True
Enabled radiomic classes	Types of descriptors extracted via PyRadiomics	First-order, GLCM, GLRLM, Shape2D, GLDM, NGTDM

**Table 3:** Poor classification performance of CNN-based models trained in the initial phase.

Model	Accuracy	AUC
EfficientNet-B1	0.52	0.6049
EfficientNet-B4	0.52	0.4214
EfficientNetV2M	0.52	0.5551
EfficientNetV2S	0.26	0.1287
ResNet50	0.27	0.1187
Xception	0.07	0.0321

**Table 4:** Paired statistical comparison of feature-selection variants across CNN models.

Model	Comparison (Best vs. Plain)	Accuracy (Percentage Points)	Paired *t*-Test	Wilcoxon	Interpretation
EfficientNet-B1	PCA (XGBoost) vs. none	+3.7	*t* = 5.15, *p* = 0.0067	W = 0, *p* = 0.062	Significant before correction; trend survives Wilcoxon
EfficientNet-B1	SKB (XGBoost) vs. none	+2.4	*t* = 2.82, *p* = 0.048	W = 0, *p* = 0.125	Non-significant after correction
EfficientNet-B4	PCA (XGBoost) vs. none	+3.0	*t* = 4.63, *p* = 0.0098	W = 0, *p* = 0.063	Significant before correction
EfficientNet-V2M	PCA (XGBoost) vs. none	+1.3	*t* = 2.40, *p* = 0.075	W = 0, *p* = 0.063	No significant change
EfficientNet-V2S	PCA (random forest) vs. none	+2.7	*t* = 6.15, *p* = 0.0035	W = 0, *p* = 0.063	Highly significant (*t* survives the Holm–Bonferroni procedure)
ResNet-50	PCA (XGBoost) vs. none	+1.7	*t* = 5.15, *p* = 0.0068	W = 0, *p* = 0.063	Significant before correction
Xception	SKB (random forest) vs. none	+0.3	*t* = 1.08, *p* = 0.34	W = 4.5, *p* = 0.50	No significant change

**Table 5:** Over-fitting checks.

Check	Rationale	Result (Mean ± SD or *p*-Value)	Interpretation
Stratified 5-fold CV (patient-level splits)	Standard internal generalization test	Baseline CNN: Acc = 0.958 ± 0.006, AUC = 0.989 ± 0.002	High but consistent scores; no single fold collapses
Paired tests (baseline vs. TTA, 5 folds)	Determines if TTA gain is by chance	Paired *t*: *t* = −4.75, *p* = 0.009 (accuracy); Wilcoxon: *p* = 0.063	Parametric test shows a modest but significant benefit; Wilcoxon borderline
200-run permutation test (labels shuffled)	Estimates probability of reaching the observed score by chance	Observed Acc = 0.968; permutation *p* = 0.005	<1% of shuffled runs match our accuracy ⇒ model is learning genuine signal
Independent 20% hold-out set	Final out-of-sample check	Acc = 0.969, AUC = 0.988; per-class F1 = 0.97	Virtually identical to CV performance ⇒ no accuracy drop-off

**Table 6:** Accuracy of each model across five feature blocks. Raw CNN = penultimate CNN embedding; Radiomics = PyRadiomics only; Fusion = CNN embedding ⊕ radiomics classified with the random forest (RF) or XGBoost; DR-ML = dimensionality-reduced CNN embedding (PCA or SelectKBest) classified with RF or XGBoost; TTA = fusion with RF plus five-view test-time augmentation. Values are mean 5-fold accuracies.

Model	Raw CNN	Radiomics	Fusion (CNN + Radiomics)	Δ Fusion-Raw (pp)	DL-ML (PCA/SKB)	TTA
EfficientNetB1	0.52	0.5584	0.6390	+12	0.6455 (PCA)	0.6477
EfficientNetB4	0.52	0.5584	0.5899	+7	0.6158 (SKB)	0.6324
EfficientNetV2M	0.52	0.5584	0.5847	+6	0.6088 (SKB)	0.5755
EfficientNetV2S	0.26	0.5584	0.8293	+57	0.8298 (SKB)	0.8490
ResNet50	0.27	0.5584	0.8000	+53	0.8087 (SKB)	0.8118
Xception	0.07	0.5584	0.9540	+88	0.9562 (SKB)	0.9628

**Table 7:** Performance of two-model fusion ensembles.

Model	Accuracy	AUC
Xception + ResNet50 + random forest + radiomics	0.9519	0.9842
Xception + ResNet50 + XGBoost + radiomics	0.9540	0.9877
Xception + EfficientNetV2S + random forest + radiomics	0.9540	0.9832
Xception + EfficientNetV2S + XGBoost + radiomics	0.9606	0.9858
Xception + EfficientNetV2S + random forest + radiomics + TTA	0.9716	0.9913
**Xception + EfficientNetV2S + XGBoost + radiomics + TTA**	**0.9716**	**0.9914**

## Data Availability

The datasets used and/or analyzed during the current study are available from the corresponding author upon reasonable request.
